# Reversion of Severe Mitral Insufficiency in Peripartum Cardiomyopathy Using Levosimendan

**DOI:** 10.14740/jocmr2323w

**Published:** 2015-10-23

**Authors:** Victor H. Nieto Estrada, Daniel L. Molano Franco, Albert Alexander Valencia Moreno, Jose A. Rojas Gambasica, Yamil E. Jaller Bornacelli, Anacaona Martinez Del Valle

**Affiliations:** a Clinica Universitaria Colombia, Bogota, Colombia; b Universidad del Rosario, Bogota, Colombia

**Keywords:** Peripartum cardiomyopathy, Levosimendan, Mitral insufficiency, Pregnancy

## Abstract

Idiopathic peripartum cardiomyopathy presenting with heart failure is a true diagnostic and treatment challenge. Goal oriented clinical management aims at the relapse of left ventricular systolic dysfunction. A 35-year-old patient on her 12th day post-delivery presents progressive signs of heart failure. Transthoracic echocardiography showed severe mitral insufficiency, mild left ventricular dysfunction, mild tricuspid insufficiency, severe pulmonary hypertension, and right atrial enlargement. With wet and cold heart failure signs, the patient was a candidate for inodilator cardiovascular support and volume depletion therapy. As the patient presented a persistent tachycardia at rest, levosimendan was chosen over dobutamine. Levosimendan was administered at a dose of 0.2 µg/kg/min during a period of 24 hours. After inodilator therapy, the patient’s signs and symptoms of heart failure began to decrease, showing improvement of dyspnea, mitral murmur grade went from IV/IV to II/IV, filling pressures and systemic and pulmonary resistance indexes decreased, arterial blood gases improved, and an echocardiography performed 72 h later showed non-dilated cardiomyopathy, mild cardiac contractile dysfunction, mild mitral insufficiency, type I diastolic dysfunction and improvement of pulmonary hypertension. Cardiovascular function in peripartum cardiomyopathy tends to go back to normality in 23-41% of the cases, but in a large group of patients, severe ventricle dysfunction remains months after initial symptoms. This article describes the diagnostic process of a patient with peripartum cardiomyopathy and a successful reversion of a severe case of mitral insufficiency using levosimendan as a new therapeutic strategy in this clinical context.

## Introduction

In 1971, Demakis and colleagues described a pathology that they called peripartum cardiomyopathy (PPCM) and established the clinical criteria for its diagnosis as follows: 1) development of heart failure in the last month of pregnancy or within 5 months of delivery; 2) absence of a determinable etiology for the cardiac failure; and 3) absence of demonstrable heart disease prior to the last month of pregnancy [1]. Further investigation added a fourth criterion: left ventricular systolic dysfunction with ejection fraction below 45% and shortening fraction below 30%, diagnosed by transthoracic echocardiography.

Recently, the European Society of Cardiology Working Group on Peripartum Cardiomyopathy proposed a definition update of PPCM as a non-familial form of peripartum heart failure characterized as an “idiopathic cardiomyopathy presenting with heart failure secondary to left ventricular systolic dysfunction towards the end of pregnancy or in the months following delivery, where no other cause of heart failure is found”. Left ventricle may not be dilated, but ejection fraction is nearly always reduced below 45% [2].

Actual incidence of PPCM in Colombia remains unknown, but there seems to be a tendency towards women older than 30 years of age, with preeclampsia history, multifetal pregnancy and African American women [3].

The etiology of this disease has been associated with multiple factors such as inflammatory processes, autoimmune reactions, inflammatory cytokines, viral infections, nutrition disorders, hormone disorders and endothelial dysfunction [4]. Recent data show that peripartum oxidative stress linked to proteolytic cleavage of prolactin into a 16 kDa subform with potent anti-angiogenic and pro-apoptotic properties may explain the heart’s microvascular damage as the onset of the myocardial disease in PPCM [5].

## Case Report

Patient is a 35-year-old female on her 12th day post-delivery via C-section with neuraxial anesthesia. She has a history of uterine myomatosis. This was her first pregnancy, with no diseases detected during complete prenatal care visits. The patient goes to the emergency room after 3 days of progressive deterioration of her functional class, complaining of sudden and severe shortness of breath, legs edema and orthopnea.

Physical examination evidenced pulse rate of 115 bpm, respiration rate of 18 bpm, blood pressure of 140/98 mm Hg, pallor, and normal cardiopulmonary auscultation.

Abdominal examination showed Pfannenstiel scar with serosanguineous drainage. The fundus of the uterus was palpable below the umbilicus. There was presence of grade II pitting edema. Neurological examination was normal.

**Figure 1:**
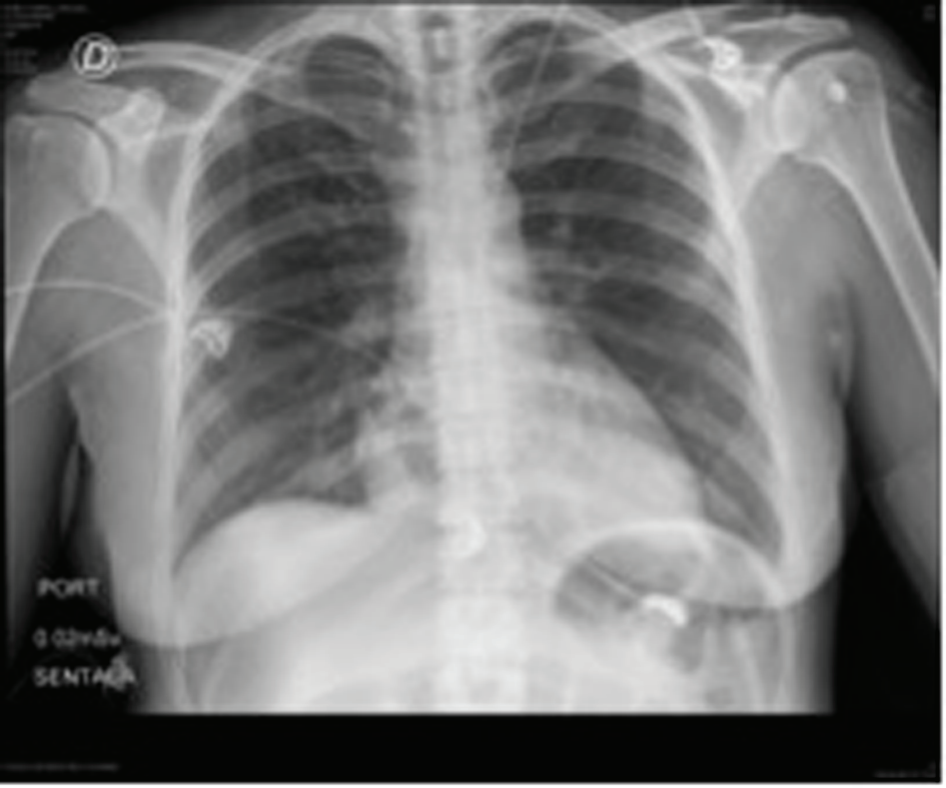
Patient’s initial chest X-rays.

Laboratories ordered during ER attention were: hemoglobin: 8.8 g/dL, hematocrit: 28%, WBC: 11,370/L, platelet count: 645,000/mL, D-dimer: 4,722 μg/dL; PT and TCT were normal. ECG revealed sinus tachycardia with repolarization alteration. Chest RX did not show any pathological changes (Fig. 1).

**Figure 2:**
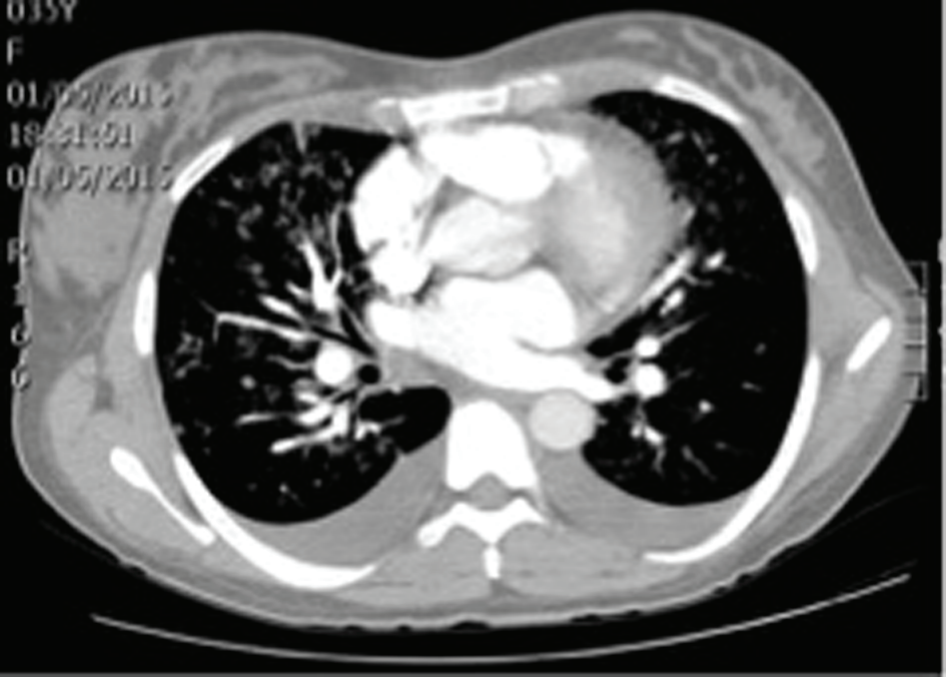
Patient’s thoracic angiotomography.

With these results in hand, the patient was clinically diagnosed with gestational hypertension, post-operatory anemia and intermediate pretest probability of pulmonary embolism. Internal medicine ordered transfusion of 2 units of packed red blood cells, a transthoracic echocardiography and thoracic angiotomography (Fig. 2).

Thoracic angiotomography ruled out pulmonary embolism but indicated bilateral pleural effusion, mild cardiomegaly and signs of venous pulmonary hypertension as indirect signs of heart failure. Transthoracic echocardiography found severe mitral insufficiency and mild left ventricular dysfunction with left ventricular ejection fraction of 47%, hypokinesis of lateral segments, mild tricuspid insufficiency, severe pulmonary hypertension (PASP 68 mm Hg), and right atrial enlargement.

These new findings supported the diagnosis of heart failure secondary to PPCM vs. acute coronary syndrome with secondary ischemic cardiomyopathy.

**Figure 3:**
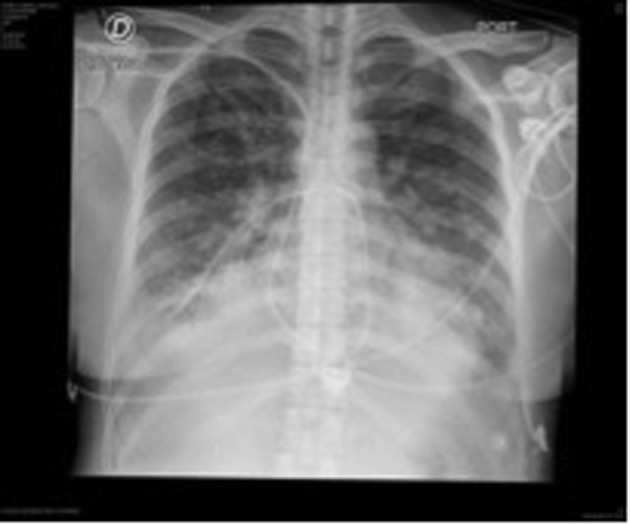
Following thoracic X-rays showing signs of pulmonary edema.

Due to torpid evolution, steadily worsening dyspnea, NT-pro BNP of 8,700 pg/mL, a positive highly sensitive troponin T and signs of pulmonary edema in new thoracic X-rays, patient was transferred to the cardiovascular intensive care unit (ICU) (Fig. 3).

Patient was severely ill at arrival at the ICU with evident signs of heart failure. She presented a systolic murmur with a S3 sound in mitral area, decreased respiratory sounds in lung bases, and hepatomegaly palpable 4 cm below the edge of the ribs. A coronary angiography was scheduled to diagnose arterial disease susceptible of percutaneous intervention, but cardiac catheterization ruled out coronary artery disease. This confirmed PPCM.

Patient’s clinical condition worsened with clinical and biochemical signs of tissue hypoperfusion, such as oliguria, hyperlactatemia, and low venous oxygen saturation. Invasive hemodynamic monitoring with Swan-Ganz catheter was initiated as soon as possible. Cardiac output was normal but filling pressures and systemic and pulmonary resistance indexes increased.

With wet and cold heart failure signs, the patient was a candidate for inodilator cardiovascular support and volume depletion therapy. As the patient presented a persistent tachycardia at rest, levosimendan was chosen over dobutamine. Levosimendan was administered at a dose of 0.2 µg/kg/min during a period of 24 h. Table 1 shows hemodynamic behavior of patient previous to levosimendan administration, 4 and 24 h after receiving levosimendan.

**Table 1:** Patient’s Hemodynamic Monitoring

Monitoring	SAP	DAP	MAP	HR	CO	IC	IS	CVP	PWP	PASP	PADP	PAMP	SVRI	PVRI
Initial	131	90	104	118	4.7	3.0	25.2	14	20	43	32	36	2,411.5	421.3
4 h after levosimendan	121	89	100	111	5.3	3.4	30.2	12	15	36	22	27	2,090.8	278.2
Post-infusion	105	69	81	106	5.8	3.7	34.6	8	10	24	12	16	1,590.9	130.8

SAP: systolic arterial pressure; DAP: diastolic arterial pressure; MAP: mean arterial pressure; HR: heart rate; CO: cardiac output; CI: cardiac index; SI: systolic index; CVP: central venous pressure; PWP: pulmonary wedge pressure; PASP: pulmonary artery systolic pressure; PADP: pulmonary artery diastolic pressure; PAMP: pulmonary artery mean pressure; SVRI: systemic vascular resistance index; PVRI: pulmonary vascular resistance index.

After inodilator therapy, the patient’s signs and symptoms of heart failure began to decrease, showing improvement of dyspnea, mitral murmur grade went from IV/IV to II/IV, filling pressures and systemic and pulmonary resistance indexes decreased, arterial blood gases improved, and an echocardiography performed 72 h later showed non-dilated cardiomyopathy with LVEF of 40%, mild cardiac contractile dysfunction, mild mitral insufficiency, type I diastolic dysfunction and improvement of pulmonary hypertension (PASP 31 mm Hg).

Discharge of ICU was decided based on diminution of heart failure symptoms, lessening of hypervolemia signs, and reversion of mitral insufficiency. Total length of stay in ICU was 5 days, patient stayed another 48 h in general ward and was discharged in good conditions with first-rate heart failure treatment and indications for ambulatory following by cardiology and gynecology.

## Discussion

PPCM represents a challenge due to the differences in the clinical cases reports and the small amount of evidence in medical literature regarding PPCM treatment [6]. This article describes in a detailed fashion the diagnostic process of a patient with PPCM and a successful reversion of a severe case of mitral insufficiency using levosimendan as a new therapeutic strategy in this clinical context.

Clinical diagnosis of PPCM can be limited because it is not clinically differentiable from any other cause of heart failure; in addition, some of its symptoms could be confused with those attributable to pregnancy, hypertension and preeclampsia [7]. For its diagnosis, the absence of previous heart disease and any other identifiable cause of heart failure is necessary; it is an exclusion diagnostic strategy.

Interpretation of biomarkers such as BNP is more difficult during pregnancy, because its levels are approximately twofold higher compared with non-pregnant women [8]. However, patients with PPCM have an additional increase triggered by the rise of the left ventricular end diastolic pressure caused by systolic dysfunction [9].

An acute myocardial infarction (MI) during pregnancy, yet infrequent, must be considered between the differential diagnosis; an MI obeys an acute coronary dissection more often than an atherothrombotic event during pregnancy [10]. This is the reason why our patient underwent coronary angiography. The use of images based diagnostic methods is essential to diagnose PPCM, including echocardiography, computed tomography and magnetic resonance imaging [11].

In terms of PPCM treatment, recommendations are based on the European Society of Cardiology guides for the management of cardiovascular diseases during pregnancy [12]. Usual heart failure treatment includes beta blockers, vasodilators and diuretics; this last group of drugs may compromise the placental perfusion and so, it requires dose adjustment to the lowest dose possible. After delivery, heart failure treatment can be optimized including ACE inhibitors, AT1 receptors antagonists or aldosterone receptor blockers [13]. There is evidence in favor of use of bromocriptine to reverse cardiogenic shock [14].

There is no formal recommendation for inotropes in pregnant patients yet. In our patient’s case, signs of shock and tissue hypoperfusion, justified the use of inotropes. The recommendation for the use of dobutamine is evidence class IIA, level C and for levosimendan is class IIB level C for heart failure in the general population [15].

The use of inotropes in PPCM and its outcomes are described mostly on case reports [16-18] and a few clinical trials with dissimilar results [19]. In the present case, the choice of levosimendan is justified by the fact that it produces a smaller chronotropic stimulus in patients with a strong adrenergic response with severe tachycardia. The result was a favorable clinical response with relapse of signs of shock and outstanding reversion of severe mitral insufficiency that permitted ICU discharge.

Available scientific literature lacks from enough strong evidence regarding PPCM. This case is evidence of the usefulness of levosimendan to treat and revert heart failure in this group of patients, demonstrated by improvements in hemodynamical variables and positive changes both clinical and echocardiographic over the myocardial structure.

Mortality associated with PPCM may vary from 0% to 19%, the need for a transplant from 6% to 11%, and cardiovascular function tends to go back to normality in 23-41% of the cases, but in a large group of patients, severe ventricle dysfunction remains months after initial symptoms [20]. Considering the natural progression of the disease, every evidence or experience that points in the direction of the founding of a guide for the treatment of PPCM is a great deal for decision making for every physician involved in the management of this kind of patients.
